# Supplementary material for: A UMLS-based spell checker for natural language processing in vaccine safety
Source: BMC Med Inform Decis Mak. 2007 Feb 12;7:3. doi: 10.1186/1472-6947-7-3 (PMC1805499; doi:10.1186/1472-6947-7-3)
Supplement: Additional file 13 — RAP application directory. Sets up directory for RAP (RDF application for PHP) source code [file 1472-6947-7-3-S13.gz › rap/doc/phpdoc/dataset/_dataset_DatasetDb_php.html]

xml version="1.0" encoding="iso-8859-1"?


Docs for page DatasetDb.php


## /dataset/DatasetDb.php

Description

Description |
Classes
| Includes

Persistent implementation of a Dataset in a database.

A RDF dataset is a collection of named RDF graphs.

History:

- 05-29-2005 : First version of this class.

- filesource: Source Code for this file
- access: public
- author: Daniel Westphal (http://www.d-westphal.de)
- author: Chris Bizer <chris@bizer.de>
- version: V0.9

Classes

Description |
Classes
| Includes

| Class | Description |
| --- | --- |
| DatasetDb | Dataset implementation. |

Includes

Description |
Classes
| Includes

require\_once
(RDFAPI\_INCLUDE\_DIR.PACKAGE\_DBASE)
(line 20)

Documentation generated on Thu, 7 Jul 2005 13:41:10 +0200 by phpDocumentor 1.3.0RC3
